# Effect of combined application of carbonate salts and hot water treatment for the management of postharvest anthracnose (*Colletotrichum gloeosporioides*) of papaya

**DOI:** 10.1002/fsn3.3366

**Published:** 2023-04-23

**Authors:** Muluken Getnet, Kumlachew Alemu, Binyam Tsedaley

**Affiliations:** ^1^ Department of Plant Science College of Agriculture and Natural Resource, Assosa University Assosa Ethiopia

**Keywords:** carbonate salts, *Colletotrichum gloeosporioides*, hot water, papaya anthracnose

## Abstract

Postharvest anthracnose (*Colletotrichum gloeosporioides Penz.Sacc*) is the most economically important biological constraint to papaya production and consumption, which causes substantial yield loss worldwide. The effect of combined application of carbonate salts and hot water treatments on the development of postharvest anthracnose and maintenances of postharvest quality of papaya fruit was studied in completely randomized design (CRD) under laboratory condition. The results revealed that combined application of hot water treatment and carbonate salts significantly (*p* < .05) reduced the incidence and severity of postharvest papaya anthracnose disease. The disease incidence reached 100% 21 days after inoculation in the control treatment; this level was significantly reduced to 26.70% by dipping the fruits in NH_4_CO_3_ at 50°C and NH_4_CO_3_ at 54°C. Similarly, treatments significantly (*p* < .05) reduced the disease severity in different degrees from the first day of disease appearance to the date of 100% unmarketability of control fruits. Furthermore, the combined application of carbonate salt and hot water treatments significantly improves fruit marketability by 93.33%. Moreover, the treatments showed significant (*p* < .05) effect on maintaining pH, TSS, TA, and reducing postharvest weight loss of papaya fruit. In conclusion, postharvest treatment of papaya fruit with NH_4_CO_3_ at 54°C, NH_4_CO_3_ at 52°C, and NaCO_3_ at 54°C can significantly reduce anthracnose development and improve marketability of the fruits without pronounced effect on their edible qualities.

## Graphical abstract

Postharvest anthracnose (*Colletotrichum gloeosporioides Penz.Sacc*) is the most economically important biological constraint to papaya production and consumption. Combined application of hot water treatment and carbonate salts significantly reduced the intensity of postharvest papaya anthracnose. Combined application of carbonate salt and hot water treatments significantly improves fruit marketability.

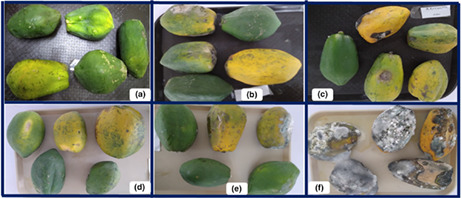

## 1 INTRODUCTION

Papaya (*Carica papaya* L.) is one of the most important fruits cultivated throughout the tropical and subtropical regions of the world. This fruit has an excellent acceptance in the global market based on its high nutritional value, lesser calories with a rich amount of dietary fiber, vitamins, and antioxidants (Tan et al., 2022). Moreover, papaya is important component of the diet in many countries in the subtropics and tropics, in regions of the world that have experienced low living standards and serious affected by malnutrition and under nutrition. Being papaya is highly nutrition fruit, it will contribute a lot in the fight against malnutrition and ensuring food security (Vij & Prashar, 2015). Papaya plants also possess medicinal and industrial value importantly used for drug development and cosmetics production (Adeyi et al., 2022; Tan et al., 2022). In spite of this fact, papaya production is affected by a number of biotic and abiotic factors, among which pre‐ and postharvest diseases are the major constraints that ultimately affect the production, utilization, and marketing (Gabrekiristos & Dagnew, 2020; Mwanauta et al., 2022).

Among the postharvest disease of papaya, anthracnose disease, which is caused by the fungus *Colletotrichum gloeosporioides Penz.Sacc*, is the most significant biological challenge to papaya production and consumption that causes substantial yield loss worldwide (Li et al., 2013; Sivakumar et al., 2002). The symptoms of anthracnose disease in papaya are characterized by round brownish depressed lesions, and in some cases, salmon‐colored areas formed by the conidial masses that cover the lesion (Ayón‐Reyna et al., 2017). Over 50% of papaya fruit produced is reported to be lost due to postharvest anthracnose disease (Torres‐Calzada et al., 2013). In Ethiopia also, papaya anthracnose is the major challenging disease of papaya fruit, which is reducing the production and quality of papaya in different parts of the country (Ademe et al., 2013).

Papaya anthracnose disease can be effectively controlled by synthetic fungicides, such as prochloraz, propiconazole, benomyl, and thiabendazole (Ali et al., 2015; Li et al., 2013; Tan et al., 2022). However, excessive use may have adverse effects on human health and the environment, accompanied by high costs, and the development of resistance by pathogen population (Gamagae et al., 2004). Moreover, the current consumer demands for organic product necessities to find alternative methods to synthetic fungicides that effectively manage the disease and maintain high fruit quality during the storage and marketing period (Vilaplana et al., 2020).

Several researches have made several efforts to develop effective postharvest disease management strategies in environmentally friendly ways to maintain high commercial quality (Li et al., 2013). Among which, hot water treatment, modified atmosphere packaging, salts, edible coating, biological control, salicylic acid plant extracts, and ozone treatment have been used to control postharvest anthracnose disease (Chan et al., 2008; de Vasconcellos Santos Batista et al., 2020; Tan et al., 2022).

Hot water treatment and carbonate salts appear to be the most promising methods used to control postharvest decay in environmentally friendly ways (Li et al., 2013; Sivakumar et al., 2002). Although hot water treatment and organic salt provide reasonable degree of control for postharvest diseases when they are used as a solo management strategy, there efficacy is not comparable to that of fungicides (Alvindia, 2013). Thus, a possible way for increasing their effectiveness is through utilization of the tactics in integrated manner.

Currently, integrated disease management is the preferred strategy because of increased concern over residual toxicity of chemical control on nontarget organisms and environment. Moreover, the limitation of a single alternative management option to achieve the same level of control and reliability as that of chemicals necessities integration of management tactics into strategies. It has been reported that hot water treatment combined with salts provides reliable and efficient level of disease control in different fruit crops (Alvindia, 2013; Ayón‐Reyna et al., 2015; Palou et al., 2001). Tan et al. (2022) also indicated the high effectiveness of the combined application of treatment for managing postharvest diseases in comparison with the single treatment while maintaining quality of the fruit. This study was conducted to determine the effect of combined application of hot water treatment and carbonate salts for the management of postharvest papaya anthracnose.

## 2 MATERIALS AND METHODS

### 2.1 Isolation of the target pathogen

The target pathogen (*C. gloeosporioides*) was isolated from papaya fruits showing anthracnose lesion using Potato Dextrose Agar (PDA) medium. An isolate of the pathogen grown in pure culture was maintained in PDA culture tubes at 4°C after conforming it pathogenicity and used as stock culture of the target organism throughout the study.

### 2.2 Experimental materials

The efficiency of carbonate salts (ammonium carbonate, calcium carbonate, and sodium carbonate) combined with hot water treatment in managing postharvest anthracnose of papaya disease was evaluated. For the purpose of the experiment, apparently healthy looking papaya fruit of local variety was marked earlier at the beginning of fruit set in the field and maintained free of chemical sprays. Undamaged, unripe, and physiologically matured fruits with comparable size and the same color class were collected directly from this tree and used for the experiments.

### 2.3 Inoculum preparation and inoculation

The fruits were harvested from the field during physiological maturity, unripe, and undamaged in the morning, and transported to Plant Pathology Laboratory. The fruits were surface‐sterilized by dipping them in a 1% sodium hypochlorite solution for 10 min. After 10 min, they were rinsed three times in sterile distilled water and air‐dried. After that, the fruits were dipped into a conidial suspension of *C. gloeosporioides* having a concentration of 10^6^ conidia/mL (Than et al., 2008).

The condial suspension of *C*. *gloeosporioides* was prepared by washing one‐to two‐week‐old cultures with sterile distilled water containing 0.01% Tween 80. Spores were suspended by gently scraping the colony's surface with a sterile loop. The suspension was transferred to a sterile test tube, and the concentration was determined using a hemocytometer and adjuster to a concentration of 10^6^ conidia/mL (Than et al., 2008).

Fruits were surface‐sterilized by dipping them in a 1% sodium hypochlorite solution for 10 min. After 10 min, they were rinsed three times in sterile distilled water and air‐dried. After that, the fruits were inoculated by dipped them into a conidial suspension of *C. gloeosporioides*. After inoculation, the fruits were covered with a plastic sheet and incubated at 25°C for 15 h until the conidia was germinate. Thereafter, the plastic covers were removed, and the fruits were dipped in a combined application of different carbonate salt solutions with hot water treatment. Papaya fruits dipped in sterile distilled water were kept separately to check if the developing disease resulted from artificial inoculation or not.

### 2.4 Combined application carbonate salts and hot water treatment for postharvest papaya anthracnose management

Inoculated papaya fruits were subjected to treatment with three types of carbonate salt, namely ammonium carbonate, calcium carbonate, and sodium carbonate, each at a concentration of 3% w/v combined with hot water temperatures (48, 50, 52, and 54°C). The fruits were treated by dipping them in hot salt solutions for 10 min in a water bath. Control fruits were dipped into sterile distilled water at room temperature. Five papaya fruits were used for each treatment, and the experiment was arranged in a completely randomized design (CRD) with three replications.

### 2.5 Disease assessment

Data on disease incidence and severity were scored for the evaluation of the combined application of carbonate salts and hot water treatment for the control of postharvest papaya anthracnose under artificial inoculation at a 48‐h interval from the time of symptom appearance to 100% of unmarketability of the control fruits. Disease incidence was measured as the percentage of papaya fruit showing any symptom of anthracnose (Ademe et al., 2013; Bautista‐Baños et al., 2002) using the following formula:

$$\begin{array}{c} \text{Disease incidence}\;\left(\mathrm{DI}\;\right)\\ =\frac{\text{Number of fruit showing anthracnose disease symptom}\;}{\text{Total number of papaya fruit assessed}}\times 100 \end{array}$$

Data on disease severity (DS) were recorded as the percentage of the fruit area covered by anthracnose lesions. Disease severity was scored by using 1 to 5 rating scale, where 1—0% of fruit area affected, 2—1%–25%, 3—26%–50%, 4—51%–75%, and 5—76%–100% of fruit area affected (Bautista‐Baños et al., 2002).

### 2.6 Postharvest quality

#### 2.6.1 Marketability of papaya fruit

Data on the proportion of marketable and unmarketable fruits were recorded at the time of disease assessment following the procedure of Dessalegn et al. (2013). Marketable fruits were determined by observing the level of visible lesion, shriveling, smoothness, and shininess of the fruit. The marketable fruits during the experiment were determined by using the following formula:

$\text{Marketability of papaya fruit}\;\left(\%\right)=\frac{\text{Number of marketable fruit}}{\text{Total number of fruit}}\times 100$

#### 2.6.2 Weight loss (WL)

Weight loss was determined as a percentage (Zhou et al., 2002) using the following formula.

$$\text{Weight loss}=\left[\left(A-B\right)/A\right]\times 100$$

where *A* is the weight of a papaya before treatment and *B* is the weight of the papaya after storage.

#### 2.6.3 Total soluble solid (TSS)

Total soluble salt was measured using a digital handheld refractometer. The TSS was determined by placing 1 to 2 drops of clear papaya juice on the prism, according to the method described previously (Wasker et al., 1991).

#### 2.6.4 Potential of hydrogen (pH)

The pH value of the papaya juice was measured with a pH meter

#### 2.6.5 Titerable acidity (TA)

Titerable acidity was analyzed from a mixture containing 5 mL of juice, 50 mL water, and 0.1% phenolphthalein, which was titrated with 0.1 M NaOH to a pH end point of 8.2. Results were expressed as a percentage of citric acid per 100 g of fresh weight using the following formula (Ali et al., 2010)

$$\mathrm{TA}=\frac{\text{Titer}\times 0.1\;\text{NaOH}\times 0.67\times 100}{100}$$

### 2.7 Data analysis

The datasets were primarily tested for various ANOVA assumptions and analyzed using SAS software version 9.4. Percentage data on disease incidence were subjected to arcsine transformation before analysis. The LSD tests were used to separate treatment means that were significantly different at 5% levels of significance. The association between disease intensity and postharvest quality parameters was computed using simple correlation analysis.

## 3 RESULTS AND DISCUSSION

### 3.1 Effect of hot water treatment and salt solution on development of anthracnose on fruit

#### 3.1.1 Effect of hot water treatment and salt solution on disease incidence

**FIGURE 1:**
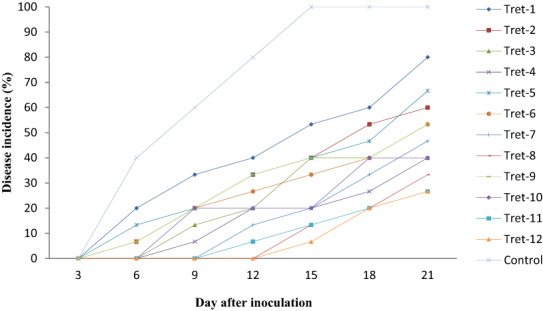
Effect of combined application of organic salt and hot water treatment on disease incidence of postharvest papaya anthracnose. Where: Tret‐1—CaCO_3_48°C, Tret‐2—CaCO_3_50°C, Tret‐3—CaCO_3_52°C, Tret‐4—CaCO_3_54°C, Tret‐5—NaCO_3_48°C, Tret‐6—NaCO_3_50°C, Tret‐7—NaCO_3_52°C, Tret‐8—NaCO_3_54°C, Tret‐9—NH_4_CO_3_48°C, Tret‐10—NH_4_CO_3_50°C, Tret‐11—NH_4_CO_3_52°C, Tret‐12—NH_4_CO_3_5°C, and Control—untreated control.

The effect of combined application of hot water treatment and salt solution significantly (*p* < .05) reduced the incidence of papaya anthracnose disease (Table 1). The disease incidence reached 100% after 21 days of inoculation in the control treatment. This level significantly reduced to 26.70% by dipping the fruits in NH_4_CO_3_ at 50°C and NH_4_CO_3_ at 54°C. Treatment of the fruit with combine application of NH_4_CO_3_ at 50°C, CaCO_3_54°C, and NaCO_3_54°C also reduced the disease incidence to a range of 33.30%–40%, which was not statistical different with the former treatments. The other treatment reduced the disease incidence with a range of 66.70%–80.00% (Table 1). The study also revealed that combined application of salts with hot water treatment significantly delayed the onset and reduced disease development in papaya fruit (Figure 1).

**TABLE 1:** Effect of combined application of organic salt and hot water treatment on disease incidence of papaya anthracnose.

Treatment	Disease incidence	Percent of disease reduction
CaCO_3_48°C	80.0 (63.4)^b^	20.0^f^
CaCO_3_50°C	60.0 (50.8)^cd^	40.0 ^de^
CaCO_3_52°C	40.0 (39.2)^efg^	60.0^abc^
CaCO_3_54°C	40.0 (39.2)^efg^	60.0^abc^
NaCO_3_48°C	66.7 (55.0)^bc^	33.3^ef^
NaCO_3_50°C	53.3 (46.9)^cde^	46.7^cde^
NaCO_3_52°C	46.7 (43.1)^def^	53.3^bcd^
NaCO_3_54°C	33.3 (35.0)^fg^	66.7^ab^
NH_4_CO_3_48°C	53.3 (46.9)^cde^	46.7^cde^
NH_4_CO_3_50°C	40.0 (39.2)^efg^	60.0^abc^
NH_4_CO_3_52°C	26.7 (30.8)^g^	73.3^a^
NH_4_CO_3_54°C	26.7 (30.8)^g^	73.3^a^
Control	100.0 (90.0)^a^	0%^g^
LSD (0.05)	8.66	14.2
CV (%)	10.9	17.3

*Note*: Incidence recorded 21 days after pathogen inoculation. Value in parenthesis is arcsine transformed. Values (mean of three replications) followed with the same latter(s) are not statistically different at *p* < .05.

The disease incidence increases sharply from its onset and reached 100% in the control 15 days after inoculation. This level was significantly reduced to 6.67% by dipping the fruit in NH4CO_2_ at 54°C. Similarly, dipping of the fruit in NH_4_CO_2_ at 52°C and NaCO_2_ at 52°C significantly reduced the disease incidence to 13.33% (Figure 1). Results of the present study demonstrated that combined application of hot water treatment and salts significantly reduced postharvest anthracnose of papaya. Similar to the present study, Ayón‐Reyan et al. (2017) indicated that combined application of hot water treatment and salts significantly delayed the appearance and reduced disease development in artificially inoculated papaya fruit.

#### 3.1.2 Effect of hot water treatment and salt solution on disease severity

**FIGURE 2:**
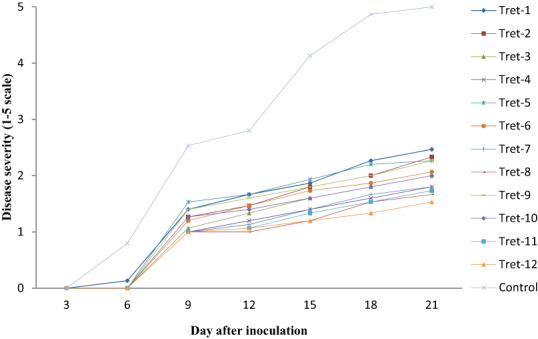
Effect of combined application of organic salt and hot water treatment on disease severity of postharvest papaya anthracnose. Where: Tret‐1—CaCO_3_48°C, Tret‐2—CaCO_3_50°C, Tret‐3—CaCO_3_52°C, Tret‐4—CaCO_3_54°C, Tret‐5—NaCO_3_48°C, Tret‐6—NaCO_3_50°C, Tret‐7—NaCO_3_52°C, Tret‐8—NaCO_3_54°C, Tret‐9—NH_4_CO_3_48°C, Tret‐10—NH_4_CO_3_50°C, Tret‐11—NH_4_CO_3_52°C, Tret‐12—NH_4_CO_3_5°C and Control = Untreated control. Disease severity assessed on a 1–5 rating scale; where: 1—no infection or spot; 2—1%–25% of the fruit Skin covered with spots/or with a few small lesions 3 = 26%–50% of the skin covered with spots with larger lesions 4—51%–75% of the skin covered with spots and 5—76%–100% of the skin covered with spots with very severe lesions.

**FIGURE 3:**
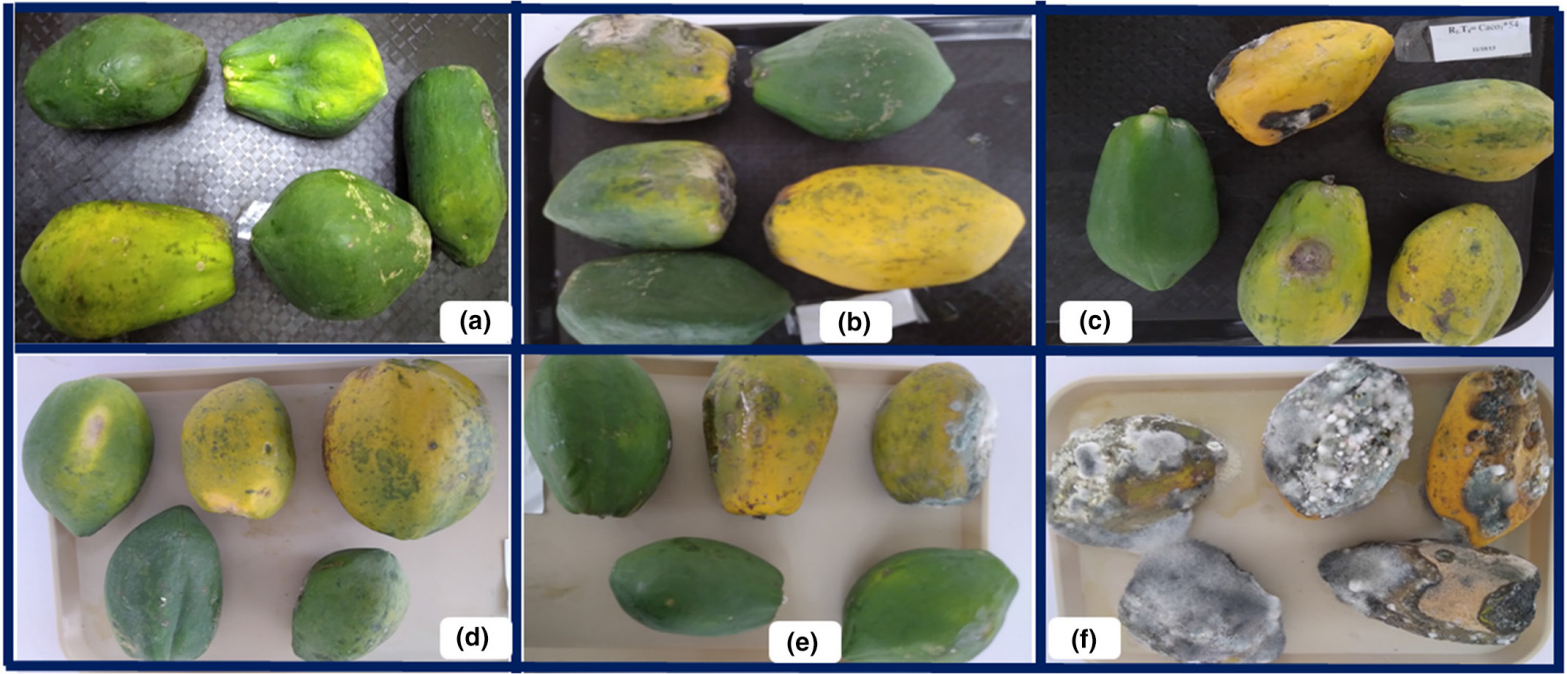
Effect of combined application of organic salt and hot water treatment on anthracnose disease severity on artificially inoculated papaya fruit 21 days after treatment. Where: (a) Fruit treated with NH_4_CO_3_ at 54°C, (b) Fruit treated NaCO_3_ at 54°C (c) Fruits treated with CaCO_3_ at 54°C and (d) Fruit treated with NH_4_CO_3_ at 52°C (e) (b) Fruit treated NaCO_3_ at 52°C (f) Untreated control.

There was significant difference (*p* < .05) among the treatments in reducing the anthracnose disease severity. All the treatments affected the disease severity in different degrees from the first day of disease appearance to the date of 100% unmarketability of control fruits (Table 2). The disease severity reached 100% in the control treatment 21 days after inoculation. This level was significantly reduced to NH_4_CO_3_ at 54°C and NaCO_3_ at 54°C with a mean severity scale of 1.53 and 1.67, respectively. Dipping of the fruit with NH_4_CO_3_ at 52°C, CaCO_3_ at 54°C, and NaCO_3_ at 52°C also significantly reduced the disease severity scale with a range of 1.73–1.80. On the contrary, combined application of salts with hot water treatment significantly delayed disease progress in artificially inoculated papaya fruit (Figure 2). The disease severity increased sharply from its onset and reached 100% in the control treatment 21 days after inoculation of the fruit. This level was significantly reduced all of the treatments. The highest disease reduction is recorded in fruit dipped with NH_4_CO_3_ at 54°C, which is statistical at par with NaCO_3_ at 54°C (Figure 2 and Figure 3).

**TABLE 2:** Effect of combined application of organic salt and hot water treatment on disease severity of papaya anthracnose.

Treatment	Disease severity
CaCO_3_48°C	2.53^b^
CaCO_3_50°C	2.33^c^
CaCO_3_52°C	1.93^de^
CaCO_3_54°C	1.73^f^
NaCO_3_48°C	2.27^c^
NaCO_3_50°C	2.07^d^
NaCO_3_52°C	1.80^ef^
NaCO_3_54°C	1.67^fg^
NH_4_CO_3_48°C	2.27^c^
NH_4_CO_3_50°C	2.00^d^
NH_4_CO_3_52°C	1.73^f^
NH_4_CO_3_54°C	1.53^g^
Control	5.00^a^
LSD (0.05)	0.17
CV (%)	4.6

*Note*: Disease severity recorded 21 days after pathogen inoculation; Disease severity assessed on a 1–5 rating scale; where: 1—no infection or spot; 2—1%–25% of the fruit skin covered with spots/or with a few small lesions 3—26%–50% of the skin covered with spots with larger lesions 4—51%–75% of the skin covered with spots, and 5—76%–100% of the skin covered with spots with very severe lesions. At final day of disease assessment Means within same columns followed by the same letter are not significantly different at *p* < .05.

The present study revealed that combined application of hot water treatment and salts significantly affects the onset and development of anthracnose disease in artificially inoculated papaya fruit. Hot water treatment of infected fruit can inhibit germination and growth of fungal pathogens established on fruit surface (Ayón‐Reyna et al., 2015; Fallik, 2004). Moreover, hot water treatment could modify the chemical environment of the fruit peel by activating the antimicrobial compounds present on it (De Costa & Erabadupitiya, 2005).

Different salts have been reported for their inhibitory and antifungal activity against different postharvest fungal pathogens (Fallik & Okon, 1996). Carbonate salts are effective in reducing fungal development probably due to their ability to elevate the pH of its exogenous environment and disruption the cellular physiology (Fallik & Okon, 1996; Palou et al., 2001). In this study, the combination of hot water and organic salts significantly reduced disease development on artificially inoculated papaya fruit. Similar results have been reported that combined application of hot water treatment and salts significantly improvement in the control activity against fungal pathogens and postharvest quality (Alvindia, 2013;Ayón‐Reyna et al., 2015). The reasons for the remarkable reduction in the development the disease could be due to the complementary effect of each single treatment (Alvindia, 2013). Ayón‐Reyna et al. (2017) indicate the combined application of hot water and salts melts the epicuticular wax, forming a physical barrier that reduces the mycelial growth and subsequent development of disease.

### 3.2 Effect of combined application of salts and hot water treatment on postharvest quality of papaya fruit

#### 3.2.1 Fruit marketability

The combined application of hot water and carbonate salt treatments showed a significant increment (*p* < .05) on fruit marketability (Tables 3). All of the fruit in the control treatment were unmarketable; 18 days after inoculation, this level was significantly improved by treatment of the fruit in combined application of hot water temperature.

**TABLE 3:** Effect of combined application of carbonate salt and hot water treatment on marketability of papaya anthracnose.

Treatment	Fruit marketability
CaCO_3_48°C	26.67^f,^ *
CaCO_3_50°C	40.00^ef^
CaCO_3_52°C	60.00^cd^
CaCO_3_54°C	66.67^cd^
NaCO_3_48°C	53.33^de^
NaCO_3_50°C	60.00^cd^
NaCO_3_52°C	73.33^bc^
NaCO_3_54°C	86.67^ab^
NH_4_CO_3_48°C	53.33^de^
NH_4_CO_3_50°C	66.67^cd^
NH_4_CO_3_52°C	86.67^ab^
NH_4_CO_3_54°C	93.33^a^
Control	0.00^g^
LSD (0.05)	16.12
CV (%)	17.33

*Note*: Values (mean of three replications) followed with the same latter(s) are not statistically different at *p* < .05.

* Marketability of papaya fruit recorded 18 days after pathogen inoculation.

The highest marketability (93.33%) recorded in fruits treated with NH_4_CO_3_ at 54°C followed by NH_4_CO_3_ at 52°C and NaCO_3_ at 54°C with a mean fruit marketability of 86.67% with no statistical difference with the former treatment, whereas dipping of the fruit in combined application of CaCO_3_ at 48°C and CaCO_3_ at 50°C showed relatively low effect in improving the marketability of papaya fruit with a mean marketability of 26.69% and 40%, respectively. Marketability of the other treatment ranged between 53.33% and 73.33%.

Decay and desiccation resulted from pathogen infection are the two most common causes of the termination of shelf life of fruits, which can be the result of various postharvest disease and other physiological disorders. The present study showed that fruit treated with combined application salt and hot water treatment showed better marketability. This may be due to the fact that the combined treatment checks the growth of microbes that are responsible for rotting and reduce metabolic rate of the fruits, which cause loss in weight through respiration (Bautista‐Baños et al., 2002). This can be demonstrated by a strong negative correlation between marketability with disease incidences (*r* = −.873) and severity (*r* = −.829) (Table 4). The elimination of incipient infections by removing spores from wounds of the fresh produce could also be a reason for increment of marketability in treated fruit. Couey (1989) and Li et al. (2013) also demonstrated that postharvest treatment of papaya fruit with hot water treatment could induce resistance to postharvest disease and effectively maintain *n* postharvest quality during storage and marketing.

**TABLE 4:** Correlation matrix of anthracnose disease intensity, marketability, and quality parameters of harvested papaya fruit.

Parameter	Incidence	Severity	Marketability	TSS	TA	pH	Weight loss
Incidence	1	+0.825**	−0.873**	+0.859**	−0.668**	−0.706**	+0.899**
Severity		1	−0.829**	+0.822**	−0.722**	−0.868**	+0.756**

Abbreviation: ns—not significant.

** Significant at *p* < .01.

#### 3.2.2 Weights loss of papaya fruit

The combined application of salt and hot water treatment significance (*p* < .05) affects weight loss of papaya fruit after harvest (Table 5); highest weight loss (10.62 g) was recorded in control treatment. However, this loss has been significantly improved by the application of carbonate salts with hot water treatment. The lowest fruit weight loss 3.94 g and 4.27 g was recorded in fruits treated with NH_4_CO_3_ 54°C and NH_4_CO_3_ 52°C, respectively. The highest weight loss in the control treatment might be due to high rate of respiration due to anthracnose infection. Similarly, Bautista‐Baños et al. (2002) indicated that high rate of infection of fruits may cause loss in weight through increased respiration rate. Moreover, Vilaplana et al. (2020) states that postharvest weight loss of papaya can be reduced with the application of postharvest treatment and reduction in papaya disease.

**TABLE 5:** Effect of combined application of organic salt and hot water treatment in maintaining postharvest quality of papaya fruit.

Treatment	Weight loss(gram)	TSS	TA	pH
CaCO_3_48°C	9.56 ± 0.04^b*^	10.80 ± 0.20^b*^	0.34 ± 0.02^c*^	5.17 ± 0.06^d*^
CaCO_3_50°C	7.88 ± 1.56^c^	10.57 ± 0.13^c^	0.35 ± 0.01^c^	5.27 ± 0.06^cd^
CaCO_3_52°C	6.04 ± 1.79^ef^	10.43 ± 0.06^c^	0.35 ± 0.01^c^	5.30 ± 0.00^b‐d^
CaCO_3_54°C	5.86 ± 1.53^f^	10.10 ± 0.09^e^	0.37 ± 0.04^bc^	5.40 ± 0.10^a‐c^
NaCO_3_48°C	7.83 ± 2.18^cd^	10.38 ± 0.16^cd^	0.35 ± 0.01^c^	5.20 ± 0.10^cd^
NaCO_3_50°C	7.68 ± 0.20^cd^	10.43 ± 0.14^c^	0.35 ± 0.00^c^	5.33 ± 0.15^b‐d^
NaCO_3_52°C	7.34 ± 1.24^cd^	10.10 ± 0.09^e^	0.42 ± 0.04^b^	5.37 ± 0.12^a‐d^
NaCO_3_54°C	5.48 ± 0.43^f^	9.85 ± 0.01^f^	0.41 ± 0.06^b^	5.57 ± 0.21^a^
NH_4_CO_3_48°C	6.93 ± 0.74^de^	10.17 ± 0.03^de^	0.37 ± 0.03^bc^	5.20 ± 0.10^cd^
NH_4_CO_3_50°C	5.28 ± 0.69^f^	10.15 ± 0.01^e^	0.38 ± 0.04^bc^	5.33 ± 0.06^b‐d^
NH_4_CO_3_52°C	4.27 ± 0.74^g^	9.77 ± 0.03^f^	0.38 ± 0.05^bc^	5.40 ± 0.10^a‐c^
NH_4_CO_3_54°C	3.94 ± 1.75^g^	9.97 ± 0.29^ef^	0.50 ± 0.05^a^	5.50 ± 0.10^ab^
Control	10.62 ± 1.01^a^	11.47 ± 0.16^a^	0.25 ± 0.03^d^	4.67 ± 0.25^e^
LSD (0.05)	0.92	0.224	0.06	0.21
CV (%)	8.07	1.30	9.10	2.30

^*^ Values (mean of three replications) followed with the same latter(s) in a column are not statistically different at *p* < .05.

#### 3.2.3 Total soluble solid

The combined application of salt solution with hot water temperature treatments showed a significant effect (*p* < .05) on maintaining total soluble soiled (TSS) harvested papaya fruit (Table 5). The highest TSS (11.47^0^Brix) was recorded in the control treatment, whereas the lowest TSS was recorded in combination of hot water treatment with salt solution at ammonium carbonate at 52°C and sodium carbonate 54°C with a mean TSS value of 9.7^0^Brix and 9.8^0^Brix, respectively. In the present study, all of the treatments were significantly better than the control treatments in maintaining TSS of harvested papaya fruit (Table 5).

The highest TSS in the control fruit may be due to increased transpiration rate as a result of high infection that accelerates ripening, resulting in faster conversion of starch to soluble sugars (Azene et al., 2014). This can be demonstrated by a strong positive correlation between TSS with disease incidences (*r* = .859) and severity (*r* = .822) (Table 4). An increase in TSS for fruits due to progressive boost in free sugars of fruit during storage periods has been reported (Brishti et al., 2013).

#### 3.2.4 Titrable acidity (TA)

The combined application of carbonate salt and hot water treatments showed a significant difference (*p* < .05) in maintaining titrable acidity of papaya fruits (Table 5). NH_4_CO_3_ at 54°C (0.50) and NaCO_3_ at 52°C (0.42) were significance effective in maintaining TA of the fruit as compared to the control treatment. The increasing in TA amount in treated papaya fruits might be attributed to the delay of respiration by treatment application. Treatment application delay respiration resulting in retardation of consumption of respiration substrates such as organic acids and sugars (Azene et al., 2014).

#### 3.2.5 Potential of hydrogen (pH)

The effect of combined application of carbonate salt and hot water treatment significantly (*p* < .05) affected pH of papaya juice (Table 5). The highest pH was recorded in NaCO_3_ at 54°C (5.57) followed by NH_4_ CO_3_ at 54°C (5.50), whereas the lowest pH was recorded in the control treatment (4.6). The variation in pH value of the juice could be due to the variation among treatments in disease intensity that ultimately affect respiration and metabolic activity of the fruits. Jitareerat et al. (2007) also suggested that the change in pH is associated with the effect of treatment on the respiration and metabolic activity of the fruits.

## 4 CONCLUSION

The effect of combined application of hot water treatment and carbonate salts significantly affected the onset and delayed the development of postharvest papaya anthracnose of papaya. Moreover, the application of NH_4_CO_3_ at 54°C, NH_4_CO_3_ at 52°C, and NaCO_3_ at 54°C for postharvest management of papaya anthracnose can significantly reduce disease development and improve marketability of papaya fruits without pronounced effect on the qualities of the fruits and could serve as a good alternative to synthetic fungicides. Further study is required on the mode of action of combined application of salt and hot water treatment, and it should be studied in more detailed to come up with concrete recommendations.

## AUTHOR CONTRIBUTIONS

**Muluken Getnet:** Conceptualization (equal); data curation (lead); formal analysis (equal); funding acquisition (lead); investigation (equal); methodology (equal); project administration (equal); resources (equal); software (lead); supervision (equal); validation (equal); visualization (equal); writing – original draft (equal); writing – review and editing (equal). **Kumlachew Alemu:** Conceptualization (equal); data curation (supporting); formal analysis (equal); funding acquisition (supporting); investigation (equal); methodology (equal); project administration (equal); resources (equal); software (lead); supervision (equal); validation (equal); visualization (equal); writing – original draft (equal); writing – review and editing (equal). **Binyam Tsedaley:** Conceptualization (equal); data curation (supporting); formal analysis (equal); funding acquisition (supporting); investigation (equal); methodology (equal); project administration (equal); resources (equal); software (supporting); supervision (equal); validation (equal); visualization (equal); writing – original draft (equal); writing – review and editing (equal).

## CONFLICT OF INTEREST STATEMENT

The authors declare that they have no conflict of interest.

## ETHICAL APPROVAL

This article does not contain any studies with human participants or animals performed by authors

## CONSENT TO PARTICIPANT

All authors read the manuscript and gave final approval for publication.

## Data Availability

The datasets used and/or analyzed during this study are available from the corresponding author upon reasonable request.
